# Focal Therapy, Differential Therapy, and Radiation Treatment for Prostate Cancer

**DOI:** 10.1155/2012/573193

**Published:** 2012-05-16

**Authors:** Anudh K. Jain, Ronald D. Ennis

**Affiliations:** Continuum Cancer Centers of New York, Department of Radiation Oncology, St. Luke's-Roosevelt Hospital Center, New York, NY 10003, USA

## Abstract

Focal and differential therapy represent an approach to improve the therapeutic ratio of prostate cancer treatments. This concept is a shift from treating the whole gland to intensely treating the portion of the gland that contains significant tumor. However, there are many challenges in the move towards focal approaches. Defining which patients are suitable candidates for focal therapy approaches is an area of significant controversy, and it is likely that additional data from imaging or detailed biopsy methods is needed in addition to traditional risk factors. A number of methods have been suggested, and imaging with multiparametric MRI and transperineal template mapping biopsy have shown promise. The approach of differential therapy where the entire prostate is treated to a lower intensity and the tumor areas to high intensity is also discussed in detail. Radiation therapy is a well suited modality for the delivery of differential therapy. Data in the literature using external beam radiation, high dose rate brachytherapy, and low-dose rate brachytherapy for differential therapy are reviewed. Preliminary results are encouraging, and larger studies and randomized controlled trials are needed to validate this approach.

## 1. Introduction

Prostate cancer is the most common malignancy in men in the US, with an estimated 241,740 cases to be diagnosed in the US in 2012 [1]. The widespread acceptance and implementation of PSA screening beginning in the early 1990s have also led to a shift to a greater number of cases diagnosed with earlier stage disease. From 1989 to 1992 the percentage of cases diagnosed with low-risk disease in the US was 29.8%, and this increased to 45.3% from 1999 to 2001 [2]. While many men with low-risk disease are well managed by surveillance [3], a large number of patients need or choose treatment. Definitive treatments such as brachytherapy, external beam radiation therapy, or radical prostatectomy generally provide high success rates with biochemical control and disease specific survival of 80–90% or higher for low-risk disease [4–6]. However, these procedures are associated with sexual, urinary, and rectal side effects that may impact patient quality of life and overall satisfaction of treatment outcome [7]. 

 Focal therapy represents an approach to improve the therapeutic ratio by maximizing tumor control while minimizing side effects. Whereas traditional therapies typically treat the entire prostate gland, this concept is a shift towards intensely treating the portion of the gland that contains significant tumor. This targeted treatment strategy has the potential to reduce the chance of injury to adjacent organs and resultant side effects while maintaining excellent oncologic outcomes. However, there are many challenges in the move towards focal therapy including identifying appropriate candidates, methodology of identifying intraprostatic tumor, and whether the remaining prostate gland should receive some form of treatment.

## 2. Candidates for Focal Approaches

Defining which patients are suitable candidates for focal therapy approaches is an area of significant controversy. Patients in the low-risk group, with the excellent outcomes described above, would appear to be the most logical choice for deintensification of treatment. Strict low-risk criteria were used by Katz et al. (Gleason < 7, PSA < 10, <T2b, only 1 biopsy core positive, no larger than 80% of a single core, no perineural invasion noted) to attempt to identify patients who could adequately be treated by focal therapy [8]. However, when 56 patients with these characteristics underwent radical prostatectomy, 12 (21%) were found to have significant bilateral secondary tumor, and thus the authors concluded that these criteria alone could not be used to select for focal therapy candidates. A consensus panel of international experts at the 2nd International Workshop on Focal Therapy and Imaging in Prostate and Kidney Cancer has issued recommendations on candidate selection for focal therapy [9]. They recommended that candidates for focal therapy should be limited to patients of low or moderate risk, and any patients with dominant Gleason 4 or clinical stage T2b or greater should be excluded. In addition, to these traditional risk factors, they also recommended that patients should undergo transperineal template mapping biopsies or multiparametric MRI with TURS biopsy. These recommendations underscore the fact that the clinician who is considering focal therapy must be able to define the anatomic location and extent of disease. As newer methods of identifying intraprostatic tumor evolve, these will be incorporated into both patient selection and treatment planning for focal therapy.

## 3. Identifying Intraprostatic Tumor

The current standard of care for prostate biopsy is to use transrectal ultrasound (TRUS) guidance to take 6–12 transrectal needle biopsies throughout the prostate in a systematic fashion. Although a reasonable approach when whole gland treatment is being considered, a comparison with prostatectomy specimens has shown that this method misses a number of clinically significant cancers and is inadequate to identify candidates for focal therapy [10]. Increasing the number of cores as in the saturation biopsy method still misses clinically significant cancer in 31% of patients [11]. Three-dimensional transperineal prostate mapping biopsy using a template grid is one way of providing a more accurate mapping of the prostate using biopsy. Preliminary studies using this method have shown good results [12], although detailed studies comparing it to radical prostatectomy are still lacking. A major drawback of this method is that it is an invasive procedure with associated complications including ecchymoses, temporary erectile dysfunction, as well as acute urinary retention [13].

Newer imaging modalities offer a noninvasive method of identifying tumor foci. Ultrasonic tissue type imaging based on spectrum analysis of radiofrequency signals has been developed by Feleppa et al., to help identify prostate cancer. When compared to biopsy data, this method has shown a receiver-operator characteristic curve (ROC) of 0.87 [14, 15]. This ultrasound imaging carries the advantage of being easily incorporated into prostate biopsies and in situ cancer treatments like brachytherapy, high-intensity ultrasound (HIFU), and cryotherapy, which are ultrasound based.

Magnetic resonance imaging (MRI), with the inclusion of diffusion and dynamic contrast, has shown even more promise in identifying intraprostatic tumors. Multiple studies have shown significant correlation between MRI abnormalities and radical prostatectomy specimens for determining size and location of cancer foci [16–19]. Cancer location and contour have been shown to have significant agreement with MRI [16, 17]. For example, in one study, MRI has been shown to have sensitivity of 86%, specificity of 94%, and receiver-operating characteristic curve of 0.874 for identifying cancer foci >0.5 mL on radical prostatectomy [18] and has in fact been shown to be accurate in detecting cancer foci as small as 0.2 cc [19]. The addition of MR spectroscopy (MRS) may improve accuracy even further, as Yamamura et al. found a sensitivity of 91.9% and specificity of 98.3% for detecting prostate cancer when using the combination of MRS and diffusion weighted imaging and comparing results to biopsy data [20]. MRS may also provide information on cancer aggressiveness, as combinations of metabolite ratios have been shown to correlate with Gleason score [21].

## 4. Limitations of Pure Focal Treatment and Rationale for Differential Therapy

A major dilemma in the implementation of focal therapy is whether the shift from treating the entire gland uniformly to purely treating the tumor only (*pure* focal therapy) is too drastic and risks missing biologically significant tumor. Prostate cancer can be multifocal in up to 87% of patients, and the prostate often contains three or more foci of cancer, and sometimes as many as twelve, on radical prostatectomy specimens [22–24]. Pretreatment risk factors have not been reliably able to predict multifocality [23], so advanced biopsy or imaging methods, as described above, are needed to define anatomic distribution of tumor. Bilaterality is also common and was seen in 80% of radical prostatectomy specimens in a series from the Duke Prostate Center, although pretreatment Gleason score and percentage of tumor involvement were found to be predictive [25]. With bilateral disease, it is doubtful that any significant gland sparing could be achieved if all tumors are adequately treated. In addition, the question of what constitutes a clinically significant tumor is a matter of controversy. Due to the long doubling time of prostate cancer, tumor size is likely to play a role in this determination. Analysis of prostate cancers, found incidentally at cystoprostatectomy by Stamey et al. suggests that cancers less than 0.5 cc are not clinically significant [26]. However Cheng et al., found 16% of cancers less than 0.5 cc found on radical prostatectomy contained Gleason pattern 4 and might, therefore, ultimately have become clinically significant [27].

Another drawback of focal therapy is that there is a lack of data on an accurate way to follow these patients. With a large portion of the gland left untreated, traditional definitions of PSA failure are not likely to be reliable. A European consensus panel suggested that oncologic efficacy would be best achieved by interval posttreatment biopsy [9]. Multiparametric MRI may also be helpful in this situation, for diagnosis or to target biopsies [28, 29]. The management of local recurrence after focal therapy also presents a challenging clinical problem—the effect of focal therapy on subsequent focal or whole gland treatments remains largely unknown.

To address the concerns of multifocality and minimize the chances of local recurrence, an alternative approach is desirable. One such approach is to decrease the intensity of the treatment to the whole prostate gland to eradicate any small foci missed by the imaging or biopsies, while simultaneously increasing the intensity to the identified tumor; an approach we term *differential* therapy. This approach, theoretically, will still improve the therapeutic ratio compared to standard whole gland treatment by decreasing the exposure of the surrounding normal tissues to the treatment.

## 5. Pure Focal Therapy

 The limited data that has been published thus far using focal therapy for prostate cancer has primarily been using HIFU, cryotherapy, and laser ablation [30–32]. Results in highly selected patients have shown excellent control rates and low toxicity, but followup has been short. All of these methods are pure focal therapies with no treatment delivered to the remainder of the prostate gland. This is an inherent limitation of these techniques, since treatment intensity is “all or nothing.” Therefore, by themselves, these treatments are not well suited for differential therapy. Instead, these therapies need to be combined with another therapy to achieve the differential therapy goal. Possible additions to these purely focal therapies include low-dose external beam radiotherapy or an oral 5-alpha reductase inhibitor.

## 6. Radiation and Differential Therapy

Radiation therapy is especially well suited to differential therapy because the treatment intensity can be varied. With modern technology and treatment planning techniques, the capability exists to deliver a high dose to the tumor region while simultaneously giving a lower dose to the remainder of the prostate gland and adjacent organs. A dose response relationship for prostate cancer has been well established for both external beam radiation therapy and brachytherapy, with higher radiation doses to the prostate resulting in higher rates of tumor control [33–36]. Likewise, a dose response relationship exists for adjacent normal tissues such as bladder, rectum, and urethra with lower doses to these organs correlating to a decreased incidence of side effects after radiation treatment with either external beam radiotherapy or brachytherapy [37–40]. These dose relationships support the hypothesis that differential therapy using radiation therapy may improve outcomes while decreasing toxicity. In addition, radiation treatment planning is image based, and the information from MRI and ultrasound for intraprostatic tumor identification can easily be incorporated to develop a treatment plan to achieve the differential therapy goals.

With the use of custom immobilization and image-guided or stereotactic methods for external beam radiotherapy, the accuracy of treatment delivery is within a few millimeters. The combination of these techniques is an exciting platform for the delivery of differential therapy with external beam radiation. This approach has thus far been explored in two ways: Miralbell et al. delivered 64 Gy external beam radiation to the entire prostate, followed by an external beam stereotactic boost of two fractions of 5–8 Gy each to the dominant tumor region of the prostate in 50 patients [41]. The tumor region was identified based on rectal examination, biopsy findings, and T2 MRI images. Treatment was delivered with a customized body cast and external markers for stereotactic guidance. They found that the technique was feasible with a 5 yr biochemical control of 98%, and 5 yr Grade 2 or greater urinary toxicity of 17.8% and rectal toxicity of 27.8%. The FLAME randomized controlled multicenter phase III trial which is ongoing in Europe is also using external beam radiotherapy for differential therapy [42]. Intermediate or high-risk patients receive 77 Gy to the entire prostate, and the experimental arm receives a simultaneous boost of 95 Gy to the area of macroscopic tumor. Multiparametric MRI is used to delineate intraprostatic tumor, and implanted fiducial markers are used for treatment set-up. The primary endpoint is to evaluate whether the addition of an “ablative microboost” to the macroscopic tumor within the prostate increases the five-year freedom from biochemical failure rate compared to the current standard of care. Secondary endpoints are treatment-related toxicity, quality of life, and disease-specific survival. 50 patients were registered as of October 2010, and accrual is ongoing.

Brachytherapy is also very well suited for differential therapy. The manual implantation of radiation sources in combination with the rapid falloff in dose as the distance from the seeds gives the physician great flexibility to vary the treatment dose to different parts of the prostate gland. In addition, there are intrinsically very high doses of radiation within a few millimeters of each source. These areas, termed “hotspots,” contain radiation doses 2-3 times the prescription dose, which allows for a creation of a higher dose gradient with brachytherapy than with external beam radiotherapy. Therefore, increasing the dose directed to the tumor while decreasing dose to the remainder of the gland with brachytherapy offers a promising method of optimizing outcomes while limiting side effects and has been used in a few preliminary studies in the literature.

High-dose-rate (HDR) brachytherapy has been used as part of a differential therapy approach, by Schick et al., to boost the dominant intraprostatic tumor in 77 patients [43]. The patients received 64 Gy external beam radiation therapy and then HDR brachytherapy to the tumor-bearing regions. Tumor was identified using information from MRI, rectal examination, and biopsy, and HDR brachytherapy was delivered with Iridium-192 source and MRI guidance. In this series, there was a subset of twenty patients who received only a boost to one side of the gland only due to presence of unilateral tumor. The five-year biochemical relapse free survival was 79.7% versus 70.5% (*P* = 0.99), for unilaterally boosted versus bilaterally boosted patients; however grade 4 toxicity was seen only in patients who had bilateral brachytherapy boost (5/57, 8.8%). The authors concluded that hemi-irradiation of the prostate with HDR brachytherapy could be considered when patients have rectal examination, MRI, and biopsy findings that suggest unilateral involvement only. Differential therapy using purely HDR brachytherapy has been investigated by Pouliot et al. at UCSF in a treatment planning study with 10 patients [44]. MRI and magnetic resonance spectroscopy (MRS) data was used to identify dominant intraprostatic lesions. HDR brachytherapy treatment plans were devised to escalate the dose to the dominant lesions while maintaining the prostate at prescription dose and minimizing doses to adjacent normal organs. They found that they could escalate the dose to the dominant intraprostatic lesion to a minimum of 120% of the prescription dose without increasing dose to adjacent organs. Further dose escalation was feasible but resulted in slightly higher doses delivered to the rectum and urethra.

Low-dose-rate (LDR) brachytherapy is also an effective method for differential radiation therapy. Todor et al. suggested dual-isotope seed implants as a technique to vary the dose throughout the prostate gland [45]. They used MRI/MRS datasets to identify disease foci within the prostate and generated nine LDR brachytherapy plans using Iodine-125, Palladium-102, and Cesium-131 alone and in combination. They found that with the combination implant plans, they were able to increase the biological effective dose to the tumor regions while achieving a reduction in dose to the urethra. Gaudet et al. performed LDR brachytherapy dose escalation to the dominant intraprostatic lesion and reported clinical results in 120 patients [46]. The location of the lesion was determined by sextant biopsy information, and the involved sextant was boosted to 150% of the prescription dose. They compared the results with 70 patients who received LDR brachytherapy with standard treatment plans. There were no differences in acute and late toxicities between the two groups. When comparing the dose escalation plans to the standard plans, they found that urethral and rectal dose parameters were lower in the group that received higher dose to the tumors. The study demonstrated the feasibility of differential therapy with LDR brachytherapy without any increase in urinary, rectal, or sexual side effects. However, the use of sextant biopsy to determine the location is limited compared to either more extensive biopsies, or imaging methods such as ultrasound spectrum analysis or MRI.

Ultrasound spectrum analysis as a method for identifying intraprostatic lesions is particularly attractive for brachytherapy. Most brachytherapy is ultrasound based, and images can be acquired at the time of the procedure and readily incorporated into the treatment plan [47]. At our institution, we are completing a phase I prospective trial to evaluate the safety and technical capability of ultrasound spectrum analysis to guide differential dose prostate LDR brachytherapy. Thus far, 14 patients have been enrolled, and we have found that the technique was feasible in all but 2 patients for a technical success rate of 86%. The tumor regions are identified on the intraoperative ultrasound spectrum analysis, and a brachytherapy treatment plan is devised to treat the tumors to 200% of the prescription dose. Standard plans were also created for each patient for comparison. Preliminary clinical and dosimetric results of the trial in the first 9 patients have also been encouraging [48]. The differential dose LDR brachytherapy plans successfully escalated the dose to the tumors compared to what would have been accomplished with a standard brachytherapy plan. A mean of 98% of the tumor volume received at least 200% of the prescribed dose compared to only 55% with the standard plans (*P* ≤ 0.0009). In addition, consistent with the previously mentioned studies, there was statistically significant reduction in dose to the prostate gland outside the tumors seen with the differential dose brachytherapy. The experimental brachytherapy has been very well tolerated, as no grade 3-4 toxicities have been noted with a followup range of 4–16 months.

## 7. Conclusion

Pure focal and differential therapy represents exciting approaches to improve the therapeutic ratio of prostate cancer treatment by decreasing complication risks while maintaining or perhaps even improving cancer control rates. Newer imaging modalities offer accurate and noninvasive methods for identifying intraprostatic tumors and can be incorporated into treatment planning. Radiation therapy is well suited for differential therapy, where treatment intensity is increased towards the tumors and decreased in the remaining prostate. Preliminary data using external beam radiotherapy, HDR brachytherapy, and LDR brachytherapy for differential therapy have been encouraging and shown that these techniques are feasible and safe. Further areas of study include defining the optimal degree of dose escalation to the tumors and dose reduction to the prostate that provide the most favorable outcomes for patients and refining the technical procedures used to deliver this dose. In addition, the possibility exists for using low-dose radiotherapy as an adjunct to HIFU, cryotherapy, and other focal treatment techniques. Additional clinical studies, and ultimately, large randomized controlled trials are needed to validate these approaches.
